# Efficacy and safety analysis of TACE + PEI + lenvatinib compared with TACE + lenvatinib for the treatment of hepatocellular carcinoma with PVTT: a retrospective study

**DOI:** 10.3389/fonc.2024.1280837

**Published:** 2024-01-17

**Authors:** Haohao Lu, Chuansheng Zheng, Bin Liang, Xiangwen Xia, Hongjie Fan

**Affiliations:** ^1^ Department of Radiology, Union Hospital, Tongji Medical College, Huazhong University of Science and Technology, Wuhan, China; ^2^ Hubei Province Key Laboratory of Molecular Imaging, Wuhan, China

**Keywords:** hepatocellular carcinoma, portal vein tumor thrombus, percutaneous ethanol injection, transarterial chemoembolization, molecular targeted therapy, complications

## Abstract

**Objective:**

The aim of this study was to investigate the efficacy and safety of transcatheter arterial chemoembolization (TACE) combined with percutaneous ethanol injection (PEI) and lenvatinib in HCC patients with PVTT (Vp2-3), thus providing a safe and effective treatment strategy for advanced HCC patients.

**Materials and methods:**

Clinical data of 227 patients with unresectable HCC and PVTT treated at the Union Hospital from January 2018 to December 2021 were retrospectively analyzed. The patients were divided into two groups according to their treatment methods: TACE+PEI+lenvatinib group (N=103) and TACE+lenvatinib group (N=124).

**Results:**

The proportion of patients with disappearance, shrinkage, or no change of PVTT after treatment was significantly higher in the TACE+PEI+lenvatinib group compared to the TACE+lenvatinib group, with statistical significance (P<0.001). The TACE+PEI+lenvatinib group had higher objective response rate (ORR) (50.5% vs. 25.8%, P<0.001) and disease control rate (DCR) (87.4% vs. 74.2%, P=0.013) than the TACE+lenvatinib group. The median progression-free survival (mPFS) of the TACE+PEI+lenvatinib group was longer than that of the TACE+lenvatinib group (8.1 months vs. 6.5 months, P<0.001). Consistently, the median overall survival (mOS) of the TACE+PEI+lenvatinib group was longer than that of the TACE+lenvatinib group (17.1 months vs. 13.9 months, P<0.001).

**Conclusion:**

Among HCC patients with PVTT (Vp2-3), TACE+PEI+lenvatinib is more effective comparing to TACE+lenvatinib in prolonging PFS and OS. The control of PVTT in the TACE+PEI+lenvatinib group was significantly more satisfactory than that in the TACE+lenvatinib group. TACE+PEI+lenvatinib is a safe and effective treatment strategy for HCC patients with PVTT (Vp2-3).

## 1 Introduction

Hepatocellular carcinoma (HCC) is one of the most common malignancies worldwide (1). According to the World Health Organization, as of 2020, the incidence of hepatocellular carcinoma ranks sixth globally, while the mortality rate ranks third (2). In some high-incidence regions such as China and Southeast Asia, the incidence and mortality rates of HCC are even higher. The high incidence of HCC is correlated with multiple factors, including viral infections, dietary habits, alcohol consumption, obesity, and exposure to drugs and toxins (3). As HCC does not show specific symptoms nor biomarkers in the early stage, many patients are already in the middle and late stages when they seek medical attention (4). Portal vein tumor thrombus (PVTT) is one of the common complications of HCC, with studies reporting that about 10%-60% of patients have PVTT (5, 6). PVTT is an important indicator of advanced-stage liver cancer, the median overall survival time is only 2-4 months for untreated patients (7). The treatment of HCC combined with PVTT has become a worldwide medical challenge, as PVTT can affect the treatment and prognosis of HCC patients (8). Once PVTT occurs in HCC patients, the staging is considered late-stage, and the choice of treatment strategy and prognosis of patients will be greatly affected (9, 10). Multiple clinical practice guidelines recommend systemic therapy as a first-line treatment for HCC with PVTT (11, 12). According to the Liver Cancer Study Group of Japan, portal vein tumor thrombus is classified into four types (Vp1-Vp4) (13), and different types of tumor thrombus have different prognoses (14). The higher the stage of PVTT, the lower the patient’s survival rate (15). Iwao Ikai et al. (16) reported a follow-up survey of more than 25,000 HCC patients who underwent surgical resection in the 18th Japanese nationwide survey. The 5-year overall survival rates of patients with Vp0, Vp1, Vp2, and Vp3/Vp4 diseases were 59.0%, 39.1%, 23.3%, and 18.3%, respectively. Transarterial chemoembolization (TACE) is one of the commonly used methods for the treatment of unresectable HCC (17). It induces tumor cell apoptosis, inhibits tumor cell proliferation by the chemotherapy drugs injected through the catheter, and at the same time, it blocks the tumor blood supply artery, leading to ischemia, hypoxia, and necrosis of tumor tissue (18). Notably, for patients in BCLC stage B, TACE can significantly improve their survival rate (19). For patients in BCLC stage C whose main portal vein is not completely occluded, TACE can also provide survival benefits (20). However, due to the dual blood supply of PVTT, TACE alone often cannot completely control the growth of the tumor thrombus. Once the tumor thrombus progresses, a series of severe complications such as liver failure, refractory ascites, gastrointestinal bleeding, and hepatic encephalopathy may occur. Therefore, a comprehensive treatment approach is often used in clinical practice for HCC combined with PVTT, which includes percutaneous ethanol injection therapy, radiotherapy, molecular targeted drugs, immunotherapy, and other treatments (21). Lenvatinib is an orally administered multi-target tyrosine kinase inhibitor that primarily inhibits VEGFR, PDGFR, and FGFR, thereby suppressing tumor angiogenesis and proliferation, with the aim of treating hepatocellular carcinoma. Additionally, Lenvatinib exerts anti-tumor effects through various pathways, such as immune modulation, inhibition of tumor cell proliferation, and induction of apoptosis (22). Therefore, for hepatocellular carcinoma patients who are not candidates for surgical resection, TACE combined with Lenvatinib has become an important treatment option (23). However, the effectiveness of TACE combined with Lenvatinib in patients with PVTT remains limited, and control of PVTT is not always achieved in some patients. After the progression of PVTT, the chance of TACE treatment may be lost, and there is also an increased risk of bleeding associated with the use of molecular targeted drugs. There is a demanding need for a direct and effective treatment for controlling PVTT in clinical practice. Percutaneous ethanol injection (PEI) is one of the commonly used local treatment methods for liver cancer. This method involves injecting anhydrous ethanol into the PVTT via a percutaneous liver puncture to destroy the tumor tissue and its blood vessels, thereby shrinking or eliminating the PVTT. Studies have explored the use of percutaneous ethanol injection in PVTT, and some have shown that it is an effective treatment method that can significantly reduce the diameter of PVTT, relieve associated symptoms, and improve patients’ quality of life (24). However, there is limited knowledge on the efficacy of TACE combined with PEI and Lenvatinib in treating advanced HCC with PVTT. The purpose of this study is to investigate the efficacy and safety of TACE combined with percutaneous ethanol injection and Lenvatinib in treating hepatocellular carcinoma with PVTT (Vp2-3) and to provide a secure and effective treatment strategy for patients with advanced hepatocellular carcinoma.

## 2 Materials and methods

### 2.1 General information

**Figure 1:**
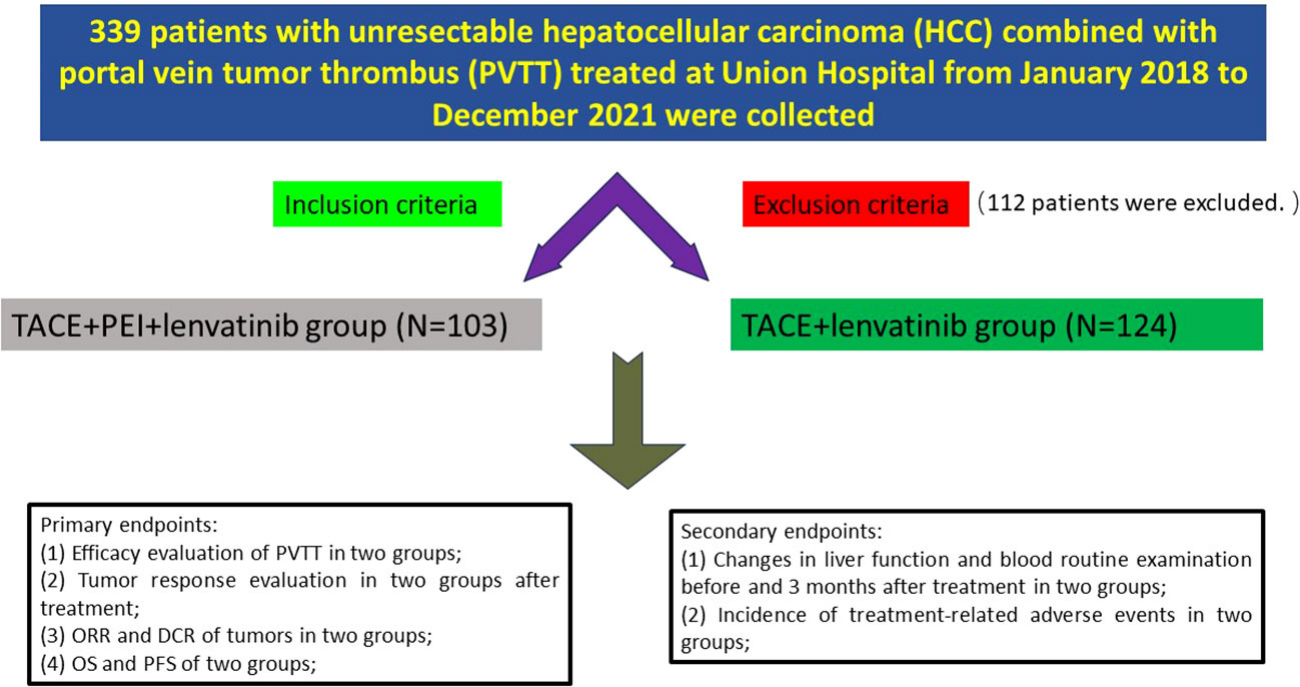
Flowchart of the study.

Clinical data of 227 patients with unresectable hepatocellular carcinoma (HCC) combined with portal vein tumor thrombus (PVTT) treated at Tongji Medical College Affiliated Union Hospital of Huazhong University of Science and Technology from January 2018 to December 2021 were collected. Inclusion criteria were: (1) age over 18 years; (2) pathological or clinical diagnosis of hepatocellular carcinoma; (3) PVTT involving the Vp2-Vp3; (4) no prior treatment for HCC, including radiation therapy, chemotherapy, or immunotherapy; (5) Child-Pugh A-B liver function classification, Eastern Cooperative Oncology Group performance status (ECOG) score of 0-2; (6) white blood cell count ≥ 3.0 G/L, platelet count ≥ 50 G/L, international normalized ratio (INR) ≤ 1.5; (7) complete clinical follow-up data. Exclusion criteria were: (1) presence of other primary or secondary cancers; (2) significant abnormalities in cardiac, pulmonary, renal, hematological, or neurological function, or coagulation function; (3) tumor volume > 70% of liver volume; (4) use of anticoagulant or antiplatelet drugs, or inability to stop these drugs; (5) massive abdominal effusion; (6) important tissue structures along the portal vein puncture pathway that cannot be avoided; (7) allergy to iodine contrast agents, ethanol, or lenvatinib. The patients were divided into two groups based on whether they received percutaneous ethanol injection (PEI): TACE + PEI + lenvatinib group (TACE + PEI + LEN group, N=103) and TACE + lenvatinib group (TACE + LEN group, N=124). (**Figure 1**) Baseline data, including gender, age, etiology of liver cirrhosis, Child-Pugh classification, ECOG score, tumor diameter, alpha-fetoprotein (AFP) level, PVTT type, total bilirubin, alanine aminotransferase (ALT), aspartate aminotransferase (AST), white blood cell (WBC) count, and platelet (PLT) count were collected.

Vp classification by the Liver Cancer Study Group of Japan (25): Vp1, PVTT confined to the portal vein distal to the second-order branch; Vp2, PVTT invading the second-order branch of the portal vein; Vp3, PVTT invading the first-order branch of the portal vein; Vp4, PVTT invading the main portal vein or contralateral first-order branch.

### 2.2 Method

#### 2.2.1 TACE Procedure

The patient lay supine with the groin area sterilized, and a sterile sheet was placed. Local anesthesia with 2% lidocaine was administered at the puncture site. The femoral artery was punctured using the Seldinger technique, and a 5F sheath was inserted. A 5F Yashiro catheter was inserted into the celiac trunk, the superior mesenteric artery, and other necessary collateral arteries to perform arterial angiography and identify the tumor-feeding arteries. Then, a 2.7F microcatheter was super-selectively inserted into the feeding artery of the hepatocellular carcinoma, and an emulsion of iodized oil and doxorubicin was injected, followed by the injection of gelatin sponge particles. If the intraoperative angiography revealed the presence of arterioportal or arteriovenous fistula, the microcatheter would be selectively advanced to the fistula site, followed by embolization using PVA particles to occlude the fistula. After that, chemoembolization would be performed. Finally, the catheter was removed, and the puncture site was compressed and bandaged.

#### 2.2.2 Usage of lenvatinib

The recommended dosage of lenvatinib is 8 mg once daily for patients with a body weight less than 60 kg and 12 mg once daily for patients with a body weight of 60 kg or greater.

#### 2.2.3 Percutaneous ethanol injection (PEI) procedure

3-5 days after TACE, under the guidance of ultrasound or CT, 2% lidocaine is used for local anesthesia at the puncture site. A sterile 21G-23G Chiba needle is percutaneously inserted into the proximal end of the portal vein tumor thrombus, and after confirming no blood backflow, anhydrous ethanol is injected into the tumor thrombus through the puncture needle, with an injection dose of 1-5 ml. PEI is repeated the next day.

Patients undergo enhanced CT or MRI every 4-6 weeks for follow-up, and subsequent TACE or PEI treatment is decided based on the follow-up results. The cut-off date for follow-up was 21 May 2023 for both groups of patients. The median follow-up period was 28.3 months in the TACE + PEI + LEN group and 26.1 months in the TACE + LEN group.

### 2.3 Observation indicators

Primary endpoints:

(1) Efficacy evaluation of portal vein tumor thrombus in two groups of patients: disappearance, shrinkage, stable or increase;
(2) Tumor response evaluation in two groups of patients after treatment, using mRECIST criteria (26), including complete response (CR), partial response (PR), stable disease (SD), and progressive disease (PD);
(3) Objective response rate (ORR) and disease control rate (DCR) of tumors in two groups of patients;
(4) Overall survival (OS) and progression-free survival (PFS) of two groups of patients;

Secondary endpoints:

(1) Changes in liver function and blood routine examination before and 3 months after treatment in two groups of patients;
(2) Incidence of treatment-related adverse events in two groups of patients;

### 2.4 Statistical methods

SPSS24.0 software was used for statistical analysis. Count data was expressed as number (percentage), and inter-group differences were tested using chi-square test, including Pearson Chi-Square and Fisher’s Exact Test. Measurement data was expressed as mean ± standard deviation, and inter-group differences were tested using t-test. OS and PFS were shown using Kaplan-Meier curves, and inter-group comparisons of OS and PFS in two groups of patients were tested using the Log-Rank test. P<0.05 indicates statistical significance.

## 3 Results

### 3.1 Comparison of baseline characteristics between the two groups

There were no significant differences in gender, etiology of liver cirrhosis, ECOG score, liver function grading, AFP level, tumor size, portal vein tumor thrombus classification, age, total bilirubin, ALT, AST, white blood cells, and platelets between the two patient groups (P>0.05) (see **Table 1**).

**Table 1:** Comparison of basic information between the two groups before treatment.

			Group				
			TACE+PEI+LEN group (N=103)	TACE+LEN group (N=124)	Chi-Square Tests(p-value)		t-test(p-value)
					Fisher’s Exact Test	Pearson Chi-Square	
Gender	Female	Count (%)	31(30.1%)	27(21.8%)	0.171	N/A	N/A
	Male	Count (%)	72(69.9%)	97(78.2%)			
Etiology of cirrhosis	Hepatitis B	Count (%)	87(84.5%%)	103(83.1%)	N/A	0.935	N/A
	Hepatitis C	Count (%)	9(8.7%)	11(8.8%)			
	others	Count (%)	7(6.8%)	10(8.1%)			
Pre-treatment ECOG	0	Count (%)	82(79.6%)	95(76.6%)	N/A	0.624	N/A
	1	Count (%)	16(15.5%)	19(15.3%)			
	2	Count (%)	5(4.9%)	10(8.1%)			
Pre-treatment liver function	Child A	Count (%)	74(71.8%)	87(70.2%)	0.883	N/A	N/A
	Child B	Count (%)	29(28.2%)	37(29.8%)			
AFP(µg/L)	<400	Count (%)	31(30.1%)	47(37.9%)	0.262	N/A	N/A
	≥400	Count (%)	72(69.9%)	77(62.1%)			
Tumor size(cm)	<5	Count (%)	45(43.7%)	61(49.2%)	0.426	N/A	N/A
	≥5	Count (%)	58(56.3%)	63(50.8%)			
Classification of portal vein tumor thrombus	VP2	Count (%)	33(32.0%)	46(37.1%)	0.485	N/A	N/A
	VP3	Count (%)	70(68.0%)	78(62.9%)			
Age (Years)	Mean ± SD		47.0 ± 12.5	48.4 ± 12.8	N/A		0.428
Pre-treatment bilirubin (μmol/L)	Mean ± SD		17.2 ± 8.5	16.5 ± 9.7	N/A		0.604
Pretreatment ALT(U/L)	Mean ± SD		43.9 ± 20.7	46.6 ± 19.2	N/A		0.309
Pretreatment AST(U/L)	Mean ± SD		48.2 ± 22.1	44.9 ± 18.2	N/A		0.215
Pretreatment WBC(G/L)	Mean ± SD		4.52 ± 1.22	4.49 ± 2.13	N/A		0.891
Pretreatment PLT(G/L)	Mean ± SD		121.78 ± 59.66	131.94 ± 70.40	N/A		0.240

### 3.2 Comparison of blood indicators three months after treatment between the two groups

There were no significant differences in total bilirubin, ALT, and PLT after treatment between the two groups (P>0.05). The TACE+LEN group had a higher AST level after treatment than the TACE+PEI+ LEN group, with statistical significance (P=0.030). The TACE+PEI+ LEN group had a higher white blood cell count after treatment than the TACE+ LEN group, with statistical significance (P=0.022) (see **Table 2**).

**Table 2:** Comparison of hematological parameters after 3 months of treatment in the two groups.

		Group		t-test(p-value)
		TACE+PEI+LEN group (N=103)	TACE+LEN group (N=124)	
Post-treatment bilirubin (μmol/L)	Mean ± SD	16.45 ± 7.76	18.26 ± 8.19	0.090
Post-treatment ALT (U/L)	Mean ± SD	54.3 ± 22.6	58.1 ± 21.9	0.201
Post-treatment AST (U/L)	Mean ± SD	51.0 ± 19.6	57.1 ± 22.0	0.030
Post-Treatment WBC (G/L)	Mean ± SD	6.16 ± 2.19	5.43 ± 2.46	0.022
Post-Treatment PLT (G/L)	Mean ± SD	87.40 ± 38.17	89.34 ± 48.77	0.737

### 3.3 Evaluation of the efficacy of portal vein tumor thrombus after treatment in the two groups

The proportion of patients in the TACE+PEI+ LEN group whose portal vein tumor thrombus disappeared, shrank, or remained stable after treatment was higher than that in the TACE+ LEN group, and the proportion of patients whose portal vein tumor thrombus increased was lower than that in the TACE+ LEN group, with statistical significance (P<0.001) (see **Table 3**).

**Table 3:** Efficacy evaluation of portal vein tumor thrombus in two groups of patients.

			Group		Chi-Square Tests(p-value)
			TACE+PEI+LEN group (N=103)	TACE+LEN group (N=124)	Pearson Chi-Square
Changes in portal vein tumor thrombus	Disappearance	Count (%)	26 (25.2%)	15 (12.1%)	<0.001
	Shrink or no change	Count (%)	61 (59.2%)	61 (49.2%)	
	enlarge	Count (%)	16 (15.6%)	48 (38.7%)	

### 3.4 Evaluation of the efficacy of tumors after treatment in the two groups 

The proportion of patients in the TACE+PEI+ LEN group who achieved CR or PR was higher than that in the TACE+ LEN group, and the proportion of patients who achieved SD or PD was lower than that in the TACE+ LEN group (P=0.001). The TACE+PEI+ LEN group had higher ORR and DCR than the TACE+ LEN group after treatment (P<0.001) (see **Table 4**).

**Table 4:** Tumor response evaluation in two groups of patients.

			Group		Chi-Square Tests(p-value)	
			TACE+PEI+LEN group (N=103)	TACE+LEN group (N=124)	Pearson Chi-Square	Fisher’s Exact Test
Tumor response	CR	Count (%)	10(9.7%)	6(4.8%)	0.001	N/A
	PR	Count (%)	42(40.8%)	26(21.0%)		
	SD	Count (%)	38(36.9%)	60(48.4%)		
	PD	Count (%)	13(12.6%)	32(25.8%)		
ORR		Count (%)	52(50.5%)	32(25.8%)	N/A	<0.001
DCR		Count (%)	90(87.4%)	92(74.2%)	N/A	0.013

### 3.5 Comparison of OS and PFS between the two groups

**Figure 2:**
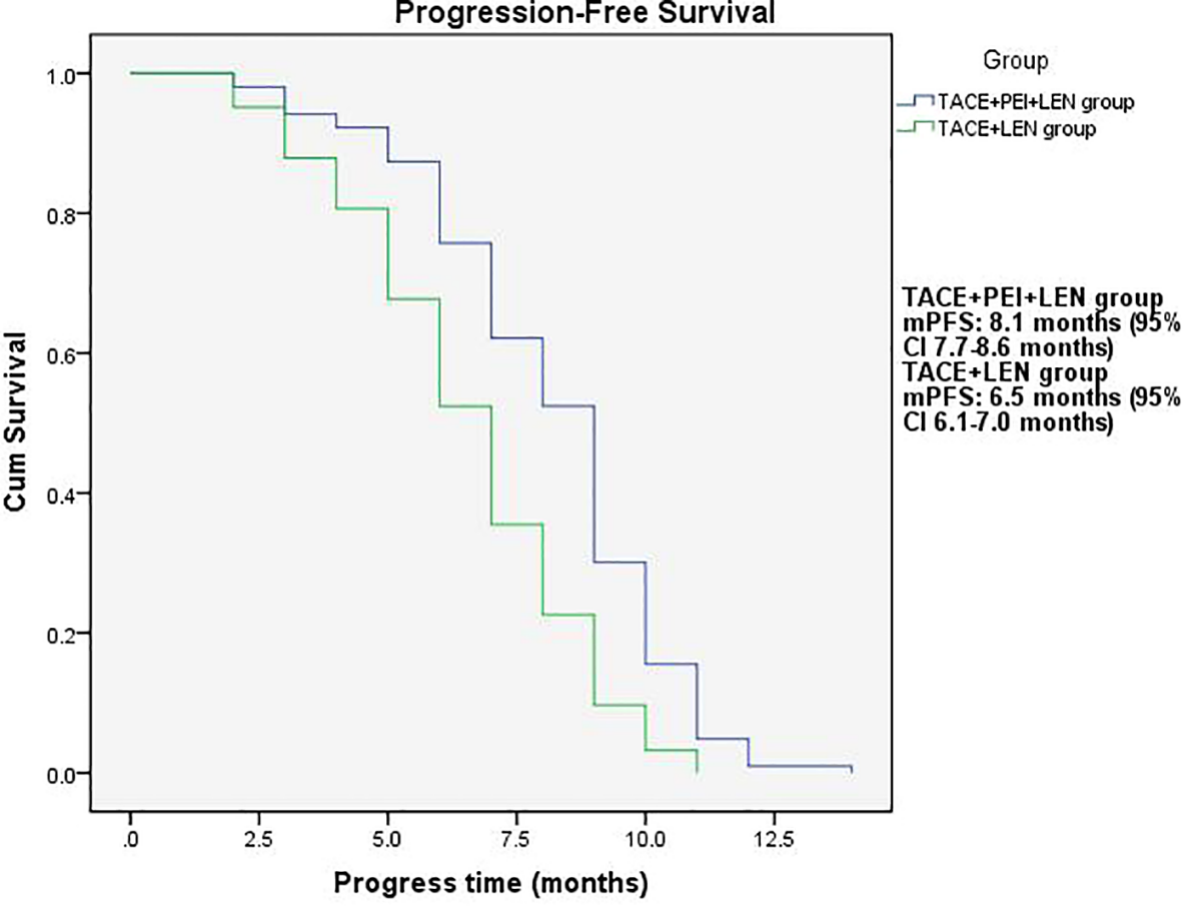
Progression-free survival time in the two groups. mPFS: TACE+PEI+LEN group, 8.1 months (95% CI: 7.7-8.6 months); TACE+LEN group, 6.5 months (95% CI: 6.1-7.0 months).

**Figure 3:**
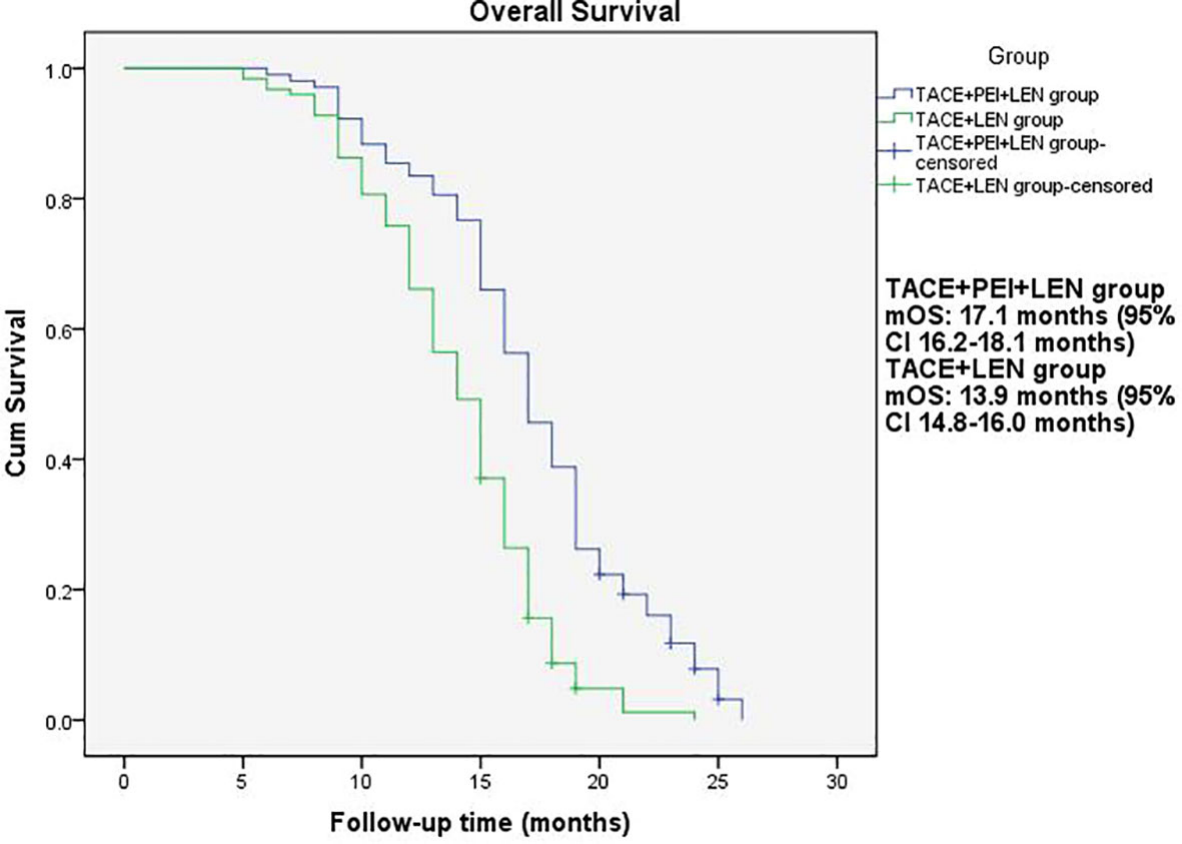
Overall survival of patients in two groups. mOS: TACE+PEI+LEN group, 17.1 months (95% CI: 16.2-18.1 months); TACE+LEN group, 13.9 months (95% CI: 14.8-16.0 months).

The median PFS in the TACE+PEI+ LEN group was longer than that in the TACE+ LEN group (8.1 months vs. 6.5 months), with statistical significance (P<0.001, **Figure 2**). The median OS in the TACE+PEI+ LEN group was longer than that in the TACE+ LEN group (17.1 months vs. 13.9 months), with statistical significance (P<0.001, **Figure 3**).

### 3.6 Incidence of treatment-related adverse events in the two groups

Incidence of Treatment-Related Adverse Events (AEs) in both groups, assessed using the Common Terminology Criteria for Adverse Events (CTCAE) version 5.0. The incidence of abdominal pain, fever, and vomiting after treatment was higher in the TACE+PEI+ LEN group than in the TACE+ LEN group, with statistical significance (P<0.05). There were no significant differences between the two groups in the incidence of hypertension, hand-foot syndrome, fatigue, and abdominal bleeding (P>0.05) (see **Table 5**).

**Table 5:** Incidence of treatment-related adverse events in two groups of patients.

	Group		Chi-Square Tests(p-value)
	TACE+PEI+LEN group(N=103)	TACE+LEN group(N=124)	
Abdominal pain	51(49.5%)	44(35.5%)	0.042
Fever	47(45.6%)	35(28.2%)	0.008
Vomiting	32(31.1%)	18(14.5%)	0.004
Hypertension	39(37.9%)	41(33.1%)	0.487
Hand-foot syndrome	46(44.7%)	54(43.5%)	0.894
Asthenia	33(32.0%)	48(38.7%)	0.331
Abdominal haemorrhage	3(2.0%)	0(0.0%)	0.092

## 4 Discussion

Hepatocellular carcinoma (HCC) is one of the most common primary malignant tumors of the liver, and its incidence and mortality rates are increasing over the years. Portal vein tumor thrombosis (PVTT) is a common complication of HCC (27).

For patients with HCC combined with PVTT who cannot undergo surgical resection, the treatment difficulty and complexity are high, and clinical treatment often cannot achieve good efficacy and prolong survival (28). Transarterial chemoembolization (TACE) has become a commonly used method for treating inoperable HCC. The theoretical basis of TACE treatment is that the normal liver tissue has dual blood supply, and the portal vein blood supply is dominant, so chemoembolization of liver tumors has a small impact on normal liver tissue. However, for patients with PVTT, if TACE is performed under the condition of portal vein blood flow obstruction, the risk of complications such as liver dysfunction, bile duct injury, liver abscess, and gastrointestinal ulceration will increase (29). Therefore, in this group of patients, TACE is relatively safe for Vp2-3 type of PVTT with the portal vein main trunk unaffected. Santhosh Anand et al. (30) reported that TACE treatment for HCC combined with PVTT without extension to the portal vein trunk can improve patient survival. Xiao Xiang et al. (31) reported on 1,040 patients with HCC combined with PVTT, of whom 675 underwent TACE treatment and 365 received best supportive care. The results showed that patients with portal vein tumor thrombosis (types I-III) who received TACE treatment had longer overall survival than those who received best supportive care. According to the BCLC guidelines, HCC patients with portal vein tumor thrombosis (PVTT) who have good liver function and physical status are classified as BCLC stage C and are recommended for systemic treatment (11). Targeted therapy is one of the commonly used systemic treatment approaches (12). Commonly used molecular targeted drugs in clinical practice include sorafenib, lenvatinib, regorafenib, and apatinib, among others (32–34). In 2017, lenvatinib was approved for first-line treatment of liver cancer based on non-inferiority to sorafenib in the REFLECT Phase III trial (23). Its efficacy has also been confirmed in clinical practice. Na Ryung Choi et al. (35) reported on 132 patients with unresectable hepatitis B-related HCC, with 44 receiving lenvatinib treatment and 88 receiving sorafenib treatment. The two groups showed no significant differences in overall survival (OS) (7.0 vs. 9.2 months, p = 0.070) and progression-free survival (PFS) (4.6 vs. 2.4 months, p = 0.134). However, the lenvatinib group had a longer time to progression (TTP) (5.2 vs. 2.5 months, p = 0.018), higher objective response rate (ORR) (18.2% vs. 4.5%, p = 0.020), and disease control rate (DCR) (77.3% vs. 47.7%, p = 0.001).

For HCC patients with PVTT, lenvatinib can also provide good survival benefits. Teiji Kuzuya et al. (36) reported on 41 patients with HCC and PVTT (Vp3/4), with 13 receiving lenvatinib treatment and 28 receiving sorafenib treatment. The lenvatinib group had higher ORR (53.8% vs. 14.3%; p = 0.0193) and DCR (92.3% vs. 35.7%; p = 0.0008) compared to the sorafenib group, and had longer median overall survival (not reached vs. 187 days; p = 0.0040). Research has reported that lenvatinib can control the progression of portal vein tumor thrombus (PVTT). Kazuhiro Takahashi et al. (37) reported a case of a patient with hepatocellular carcinoma and PVTT (Vp4) where the tumor thrombus involved the main portal vein and contralateral third-order branches. After three months of treatment with lenvatinib, the PVTT significantly regressed, and the patient underwent surgical treatment, with postoperative pathology indicating that most of the PVTT had necrosed. The results of this study showed that the percentage of patients with disappearing, shrinking, or stable PVTT in the TACE + lenvatinib group was 61.3%.

However, TACE poses many challenges in treating PVTT because PVTT often has dual blood supplies, is directly located in the portal vein system, and the feeding arteries of the thrombus cannot be completely blocked to cut off the nutrient supply. In addition, the feeding arteries of PVTT are often small and complex, making it difficult to control the growth of PVTT by TACE (38). Studies have reported that TACE can lead to ischemia and hypoxia in tumor tissue, which upregulates hypoxia-inducible factor-1 alpha (HIF-1α) and induces the upregulation of VEGF, PDGF, FGF, and other factors. This may lead to tumor recurrence and metastasis (39, 40). Lenvatinib inhibits targets such as VEGFR, PDGFR, and FGFR and thus has a synergistic effect when used in combination with TACE (41). Biao Yang et al. (42) reported that TACE combined with lenvatinib was superior to TACE combined with sorafenib in terms of OS, PFS, and ORR for HCC patients with PVTT, with mOS of 16.4 months versus 12.7 months, mPFS of 8.4 months versus 7.43 months, and ORR of 66.8% versus 33.3%. Xiaoyan Ding et al. (43) reported that TACE combined with lenvatinib was safe, well-tolerated, and effective for late-stage liver cancer with PVTT compared to TACE combined with sorafenib, with longer mTTP (4.7 vs. 3.1 months) and higher ORR (53.1% vs. 25.0%). Zhenwei Peng et al. (44) reported that TACE combined with lenvatinib had a longer mOS (17.8 vs. 11.5 months), mPFS (10.6 vs. 6.4 months), and higher ORR (54.1% vs. 25.0%) compared to lenvatinib alone in 338 patients with advanced HCC. Zhigang Fu et al. (45) reported that TACE combined with lenvatinib significantly improved the one-year (88.4% vs. 79.2%) and two-year (79.8% vs. 49.2%) OS rates compared to TACE alone and had a higher ORR (68.3% vs. 31.7%, p < 0.001). The results of this study showed that the ORR of the TACE + lenvatinib group was 25.8%, and the DCR was 74.2%, with mOS of 13.9 months.

Percutaneous ethanol injection (PEI) is a chemical ablation technique that involves injecting ethanol into the target area after percutaneous puncture of the skin. Ethanol causes dehydration and protein denaturation in tissues, leading to tissue inactivation (46). It is commonly used for the treatment of small liver lesions and has shown good therapeutic efficacy in clinical practice (47), especially for high-risk special sites, such as those close to the diaphragm, large blood vessels, bile ducts, intestinal tracts, or liver capsule (48). Shuichiro Shiina et al. (49) reported on 685 patients with hepatocellular carcinoma (HCC) who underwent 2,147 PEI treatments, with complete tumor ablation achieved in 2,108 treatments (98.2%). The median follow-up time was 51.6 months, and the 5-year, 10-year, and 20-year survival rates were 49.0%, 17.9%, and 7.2%, respectively. Masaaki Ebara et al. (50) studied 270 patients with small liver cancer who underwent PEI treatment and found that all HCC cases were completely resolved by PEI, with no treatment-related deaths and only 2.2% experiencing severe complications, leading to the conclusion that PEI is a reliable method for treating small HCC. PEI has also been used to treat portal vein tumor thrombosis. Usually, the puncture needle is inserted into the “head” or proximal end of the portal vein tumor thrombus, and then the anhydrous ethanol is injected. However, there are differences in the amount and frequency of anhydrous ethanol injection among different centers. T Livraghi et al. (51) reported 4 cases of HCC combined with portal vein tumor thrombus treated with percutaneous ethanol injection, in which one patient had complete necrosis of the tumor thrombus, two patients had no progression of the tumor thrombus during follow-up of 4-12 months, and one patient had significant reduction of the tumor thrombus and maintained it for 13 months. Previous research has shown that PEI can control tumor thrombus progression. Due to the effectiveness of PEI, this study used TACE+PEI+ lenvatinib to treat HCC patients with portal vein tumor thrombosis (Vp2-3) in order to better control portal vein tumor thrombosis and provide patients with more treatment options and better survival benefits. The results showed that the proportion of patients in the TACE+PEI+ LEN group with disappearance, shrinkage or stabilization of portal vein tumor thrombosis was 84.4%, which was significantly higher than that in the TACE+ LEN group, with a statistically significant difference (P<0.001). The TACE+PEI+ LEN group had higher ORR (50.5% vs 25.8%, P<0.001) and DCR (87.4% vs 74.2%, P=0.013) than the TACE+ LEN group after treatment. The mPFS of the TACE+PEI+ LEN group was 8.1 months, while that of the TACE+ LEN group was 6.5 months; the mOS of the TACE+PEI+ LEN group was 17.1 months, while that of the TACE+ LEN group was 13.9 months; both had statistical differences, with P<0.001(**Figures 2**, **3**).

In this study, a follow-up blood test at three months after treatment showed that AST levels were higher in the TACE+ LEN group than in the TACE+PEI+ LEN group. The possible reason for this difference may be due to poorer control of intrahepatic tumors and portal vein thrombosis in the TACE+ LEN group, leading to decreased normal liver tissue and reduced portal vein perfusion. Among the main treatment-related adverse events, the TACE+PEI+ LEN group had a higher incidence of abdominal pain (49.5% vs 35.5%), fever (45.6% vs 28.2%), and vomiting (31.1% vs 14.5%) than the TACE+ LEN group (P<0.05). Post-embolization syndrome is a common adverse reaction after TACE, which includes abdominal pain, fever, and nausea/vomiting, and can be relieved by symptomatic treatment in the short term (52, 53). PEI is a commonly used method of chemical ablation, and like other ablation therapies, it may cause abdominal bleeding due to liver puncture. The inflammatory reaction caused by tumor tissue necrosis can lead to pain and fever in patients. PEI can also cause damage to the bile duct or liver abscess. The high concentration of ethanol injected during PEI can also cause pain and vomiting. If a large amount of ethanol is injected, patients may experience drunkenness and other reactions. According to a study by N. Elgindy et al. (54), complications of high-dose ethanol injection for the treatment of liver tumors include pain, syncopation, nausea/vomiting, sepsis, and abscess. In this study, the incidence of abdominal pain, fever, and vomiting in the TACE+PEI+ LEN group was higher than that in the TACE+ LEN group, possibly due to the fact that patients in the former group received two types of local treatment in a short period of time. After close observation and symptomatic treatment, all patients experienced symptom relief without any serious complications.

PEI involves percutaneous transhepatic puncture of the portal vein using a puncture needle, and as all patients in our study had concomitant liver cirrhosis and portal hypertension, with some having Child-Pugh B liver function, the risk of bleeding during puncture cannot be ignored. In a report by M Di Stasi et al. (55), among 1,066 patients with hepatocellular carcinoma who underwent 8,118 sessions of PEI, eight patients (0.7%) experienced bleeding. However, the Chiba needle we chose for puncture was very thin (21G-23G), and all our patients underwent puncture under ultrasound or CT guidance, with the selected puncture pathway passing through normal liver tissue without important blood vessels or other structures. Therefore, none of our patients experienced significant bleeding. In the TACE+PEI+ LEN group, three patients (2.0%) experienced a small amount of bleeding around the liver in the abdominal cavity, but their condition stabilized after bed rest and use of hemostatic drugs, and they were discharged without any signs of increased bleeding. Although the incidence of abdominal bleeding was slightly higher in the TACE+PEI+ LEN group than in the TACE+ LEN group, there was no statistically significant difference between the two groups (P=0.092).

The limitations of this study include the fact that the data comes from a single center and that it is a retrospective study with a limited sample size. In the future, multi-center, large-sample prospective studies will be conducted, which will provide more insights for clinical work.

## 5 Conclusion

For patients with hepatocellular carcinoma (HCC) and portal vein tumor thrombus (PVTT) (Vp2-3), TACE+PEI+lenvatinib significantly prolongs both progression-free survival (mPFS, 8.1 months vs 6.5 months) and overall survival (mOS, 17.1 months vs 13.9 months) compared to TACE+lenvatinib. The control of PVTT is significantly better in the TACE+PEI+lenvatinib group (<0.001). Although the incidence of abdominal pain, fever, and vomiting is higher in the TACE+PEI+lenvatinib group than in the TACE+lenvatinib group (P<0.05), the symptoms are managable and do not affect the overall treatment of the patients. TACE+PEI+lenvatinib is a safe and effective treatment strategy for patients with HCC and PVTT (Vp2-3).

## Data availability statement

The raw data supporting the conclusions of this article will be made available by the authors, without undue reservation.

## Ethics statement

The studies involving humans were approved by The Ethics Committee of Union Hospital, Tongji Medical College, Huazhong University of Science and Technology. The studies were conducted in accordance with the local legislation and institutional requirements. Written informed consent for participation was not required from the participants or the participants’ legal guardians/next of kin in accordance with the national legislation and institutional requirements.

## Author contributions

HL: Investigation, Writing – original draft, Writing – review & editing. CZ: Conceptualization, Data curation, Investigation, Writing – review & editing. BL: Data curation, Formal analysis, Investigation, Methodology, Writing – original draft. XX: Data curation, Investigation, Methodology, Writing – original draft, Writing – review & editing. HF: Data curation, Formal analysis, Investigation, Methodology, Writing – original draft.
